# 1-(2-Oxoindolin-3-yl­idene)-4-[2-(tri­fluoro­meth­yl)phen­yl]thio­semicarbazide

**DOI:** 10.1107/S160053681003343X

**Published:** 2010-08-25

**Authors:** Muhammad Ramzan, Humayun Pervez, Muhammad Yaqub, M. Nawaz Tahir

**Affiliations:** a Department of Chemistry, Bahauddin Zakariya University, Multan 60800, Pakistan; b Department of Physics, University of Sargodha, Sargodha, Pakistan

## Abstract

In the title compound, C_16_H_11_F_3_N_4_OS, the dihedral angle between the aromatic ring systems is 69.15 (10)°. Intra­molecular N—H⋯N and N—H⋯O hydrogen bonds generate *S*(5) and *S*(6) rings, respectively. A short N—H⋯F contact also occurs. In the crystal, inversion dimers linked by pairs of N—H⋯O hydrogen bonds generate *R*_2_^2^(8) loops. The dimers are linked by N—H⋯F hydrogen bonds, forming polymeric chains propagating in [100]. π–π inter­actions also exist between the centroids of the benzene rings of the 2-oxoindoline group at a distance of 3.543 (3) Å and a short C=O⋯π contact occurs. Two F atoms of the trifluoro­methyl group are disordered over two sets of sites in a 0.517 (8):0.483 (8) ratio.

## Related literature

For the synthetic and biological background see: Pervez *et al.* (2007 ▶, 2008 ▶, 2010*a* ▶). For a related structure, see: Pervez *et al.* (2010*b* ▶). For graph-set notation, see: Bernstein *et al.* (1995 ▶).
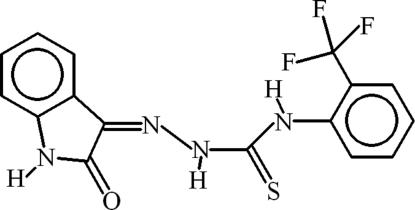


## Experimental

### 

#### Crystal data

C_16_H_11_F_3_N_4_OS
*M* *_r_* = 364.35
Monoclinic, 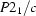

*a* = 4.5214 (3) Å
*b* = 16.6197 (14) Å
*c* = 21.6111 (18) Å
β = 93.241 (3)°
*V* = 1621.4 (2) Å^3^
*Z* = 4
Mo *K*α radiation
μ = 0.24 mm^−1^
*T* = 296 K
0.32 × 0.14 × 0.12 mm

#### Data collection

Bruker Kappa APEXII CCD diffractometer
Absorption correction: multi-scan (*SADABS*; Bruker, 2005 ▶) *T*_min_ = 0.962, *T*_max_ = 0.970
12183 measured reflections
2886 independent reflections
1920 reflections with *I* > 2σ(*I*)
*R*_int_ = 0.042

#### Refinement

*R*[*F* ^2^ > 2σ(*F* ^2^)] = 0.055
*wR*(*F* ^2^) = 0.167
*S* = 1.02
2886 reflections
227 parameters
H-atom parameters constrained
Δρ_max_ = 0.81 e Å^−3^
Δρ_min_ = −0.42 e Å^−3^

### 

Data collection: *APEX2* (Bruker, 2007 ▶); cell refinement: *SAINT* (Bruker, 2007 ▶); data reduction: *SAINT*; program(s) used to solve structure: *SHELXS97* (Sheldrick, 2008 ▶); program(s) used to refine structure: *SHELXL97* (Sheldrick, 2008 ▶); molecular graphics: *ORTEP-3* (Farrugia, 1997 ▶) and *PLATON* (Spek, 2009 ▶); software used to prepare material for publication: *WinGX* (Farrugia, 1999 ▶) and *PLATON*.

## Supplementary Material

Crystal structure: contains datablocks global, I. DOI: 10.1107/S160053681003343X/hb5611sup1.cif

Structure factors: contains datablocks I. DOI: 10.1107/S160053681003343X/hb5611Isup2.hkl

Additional supplementary materials:  crystallographic information; 3D view; checkCIF report

## Figures and Tables

**Table 1:** Hydrogen-bond geometry (Å, °) *Cg*1 is the centroid of the C1/C2/C7/N1/C8 ring.

*D*—H⋯*A*	*D*—H	H⋯*A*	*D*⋯*A*	*D*—H⋯*A*
N1—H1⋯O1^i^	0.86	2.03	2.864 (5)	163
N3—H3*A*⋯O1	0.86	2.09	2.766 (4)	135
N4—H4*A*⋯F1^ii^	0.86	2.24	2.998 (5)	147
N4—H4*A*⋯F2*A*	0.86	2.39	2.745 (9)	105
N4—H4*A*⋯N2	0.86	2.16	2.582 (5)	110
C3—H3⋯F3*A*^iii^	0.93	2.48	3.017 (10)	117
C8—O1⋯*Cg*1^iv^	1.23 (1)	3.42 (1)	3.835 (5)	100 (1)

Symmetry codes: (i) 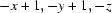; (ii) 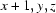; (iii) 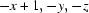; (iv) 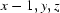.

**Fig. 1.**
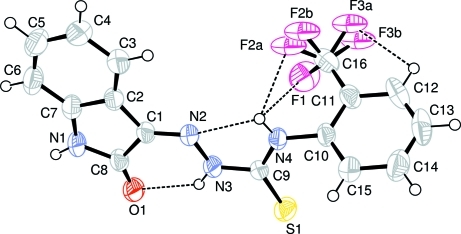
View of (I) with displacement ellipsoids drawn at the 50% probability level. H-atoms are shown by small circles of arbitrary radius.

**Fig. 2.**
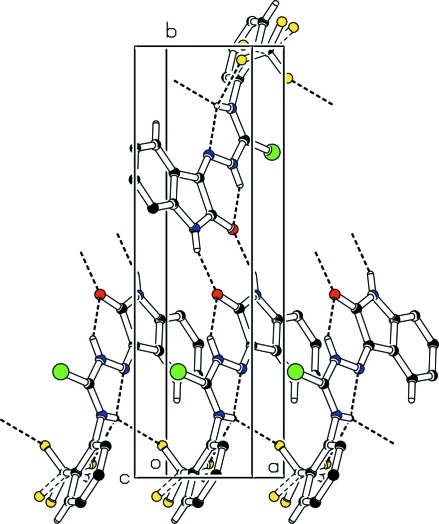
The partial packing of (I), which shows that molecules form one-dimensional polymeric chains extending along the a axis due to interlinkage of dimers with different ring motifs.

## supplementary crystallographic information

### Comment

In continuation of our work on the synthesis of biologically important isatin derivatives (Pervez *et al.*, 2007, 2008, 2010*a*), we report herein the structure and synthesis of the title compound (I, Fig. 1).

The crystal structure of (II) *i.e.* 4-(2-fluorophenyl)-1-(2-oxoindolin-3-ylidene)thiosemicarbazide has been published (Pervez *et al.*, 2010*b*). The title compound (I) differs from (II) due to the presence of trifluoromethyl instead of fluoro function at position-2 of the phenyl ring substituted at N^4^ of the thiosemicarbazone moiety.

In (I), the 2-oxoindolin A (C1–C8/N1/O1), thiosemicarbazide B (N2/N3/C9/S1/N4) and the phenyl ring C (C10—C15) of the trifluoromethylphenyl substituant are almost planar with r. m. s. deviations of 0.0243, 0.0199 and 0.0035 Å, respectively. The dihedral angle between A/B, A/C and B/C is 5.56 (16)°, 69.15 (10)° and 68.08 (11)°, respectively. Due to intramolecular H-bondings (Table 1, Fig. 1), two S(5) and three S(6) (Bernstein *et al.*, 1995) ring motifs are formed. The molecules are dimerized (Fig. 2) due to intermolecular H-bonding of N—H···O type with *R*_2_^2^(8) ring motifs. There exist C═O···π interaction (Table 1). There also exist π–π interactions between the centroids of the rings of 2-oxoindolin at a distance of 3.543 (3) Å. The dimers are interlinked from the ends through N—H···F type of H-bondings. The interlinkage of dimers make one dimensional polymeric chains extending along the *a* axis. The trifluoromethyl substituant has two F-atoms disordered over two set of sites with occupancy ratio of 0.517 (8):0.483 (8).

### Experimental

To a hot solution of isatin (0.74 g, 5.0 mmol) in ethanol (10 ml) containing a few drops of glacial acetic acid was added 4-(2-(trifluoromethyl)phenyl)thiosemicarbazide (1.18 g, 5.0 mmol) dissolved in ethanol (10 ml) under stirring. The reaction mixture was then heated under reflux for 2 h. The yellow crystalline solid formed during heating was collected by suction filtration. Thorough washing with hot ethanol followed by ether furnished the target compound in pure form (1.37 g, 83%), m.p. 529 K (*d*). The dark yellow needle-like crystals of (I) were grown in ethyl acetate by slow evaporation at room temperature.

### Refinement

The trifluoromethyl substituent has two F-atoms disordered. The disordered atoms were treated with equal anisotropic thermal parameters which resulted in the occupancy ratio of 0.517 (8):0.483 (8).

The H-atoms were positioned geometrically (N–H = 0.86 Å, C–H = 0.93 Å) and refined as riding with *U*_iso_(H) = *xU*_eq_(C, N), where *x* = 1.2 for all other H-atoms.

### Crystal data

**Table:**

C_16_H_11_F_3_N_4_OS	*F*(000) = 744
*M**_r_* = 364.35	*D*_x_ = 1.493 Mg m^−^^3^
Monoclinic, *P*2_1_/*c*	Mo *K*α radiation, λ = 0.71073 Å
Hall symbol: -P 2ybc	Cell parameters from 1920 reflections
*a* = 4.5214 (3) Å	θ = 2.3–25.1°
*b* = 16.6197 (14) Å	µ = 0.24 mm^−^^1^
*c* = 21.6111 (18) Å	*T* = 296 K
β = 93.241 (3)°	Needle, yellow
*V* = 1621.4 (2) Å^3^	0.32 × 0.14 × 0.12 mm
*Z* = 4	

### Data collection

**Table:**

Bruker Kappa APEXII CCD diffractometer	2886 independent reflections
Radiation source: fine-focus sealed tube	1920 reflections with *I* > 2σ(*I*)
graphite	*R*_int_ = 0.042
Detector resolution: 8.3 pixels mm^-1^	θ_max_ = 25.1°, θ_min_ = 2.3°
ω scans	*h* = −5→3
Absorption correction: multi-scan (*SADABS*; Bruker, 2005)	*k* = −19→19
*T*_min_ = 0.962, *T*_max_ = 0.970	*l* = −25→25
12183 measured reflections	

### Refinement

**Table:**

Refinement on *F*^2^	Primary atom site location: structure-invariant direct methods
Least-squares matrix: full	Secondary atom site location: difference Fourier map
*R*[*F*^2^ > 2σ(*F*^2^)] = 0.055	Hydrogen site location: inferred from neighbouring sites
*wR*(*F*^2^) = 0.167	H-atom parameters constrained
*S* = 1.02	*w* = 1/[σ^2^(*F*_o_^2^) + (0.076*P*)^2^ + 1.5855*P*] where *P* = (*F*_o_^2^ + 2*F*_c_^2^)/3
2886 reflections	(Δ/σ)_max_ < 0.001
227 parameters	Δρ_max_ = 0.81 e Å^−^^3^
0 restraints	Δρ_min_ = −0.42 e Å^−^^3^

### Special details

**Table:**

Geometry. Bond distances, angles *etc*. have been calculated using the rounded fractional coordinates. All su's are estimated from the variances of the (full) variance-covariance matrix. The cell e.s.d.'s are taken into account in the estimation of distances, angles and torsion angles
Refinement. Refinement of *F*^2^ against ALL reflections. The weighted *R*-factor *wR* and goodness of fit *S* are based on *F*^2^, conventional *R*-factors *R* are based on *F*, with *F* set to zero for negative *F*^2^. The threshold expression of *F*^2^ > σ(*F*^2^) is used only for calculating *R*-factors(gt) *etc*. and is not relevant to the choice of reflections for refinement. *R*-factors based on *F*^2^ are statistically about twice as large as those based on *F*, and *R*- factors based on ALL data will be even larger.

### Fractional atomic coordinates and isotropic or equivalent isotropic displacement parameters (Å^2^)

**Table:**

	*x*	*y*	*z*	*U*_iso_*/*U*_eq_	Occ. (<1)
S1	0.1580 (3)	0.24551 (7)	0.21357 (6)	0.0553 (4)	
F1	−0.0115 (7)	0.0741 (2)	0.08290 (15)	0.0825 (12)	
F2A	0.375 (2)	0.0318 (5)	0.0597 (4)	0.0788 (14)	0.517 (8)
F3A	0.0360 (19)	−0.0568 (5)	0.0737 (4)	0.0788 (14)	0.517 (8)
O1	0.4521 (8)	0.42453 (18)	0.06431 (14)	0.0553 (10)	
N1	0.7546 (8)	0.4227 (2)	−0.01834 (16)	0.0499 (11)	
N2	0.6597 (8)	0.25246 (19)	0.07503 (15)	0.0416 (11)	
N3	0.4709 (8)	0.27331 (19)	0.11784 (15)	0.0430 (11)	
N4	0.4895 (8)	0.14419 (19)	0.15100 (16)	0.0466 (11)	
C1	0.7346 (9)	0.3061 (2)	0.03602 (18)	0.0394 (12)	
C2	0.9279 (9)	0.2941 (3)	−0.01449 (18)	0.0427 (12)	
C3	1.0847 (11)	0.2284 (3)	−0.0344 (2)	0.0543 (17)	
C4	1.2404 (12)	0.2366 (3)	−0.0870 (2)	0.0645 (17)	
C5	1.2403 (12)	0.3090 (4)	−0.1191 (2)	0.0662 (19)	
C6	1.0873 (11)	0.3748 (3)	−0.0996 (2)	0.0619 (17)	
C7	0.9314 (10)	0.3661 (3)	−0.04719 (19)	0.0458 (14)	
C8	0.6269 (10)	0.3914 (3)	0.03103 (19)	0.0438 (14)	
C9	0.3807 (9)	0.2175 (2)	0.15997 (17)	0.0377 (12)	
C10	0.4371 (9)	0.0756 (2)	0.18801 (19)	0.0428 (14)	
C11	0.2761 (10)	0.0108 (3)	0.1631 (2)	0.0491 (16)	
C12	0.2286 (12)	−0.0553 (3)	0.2002 (3)	0.070 (2)	
C13	0.3394 (14)	−0.0571 (3)	0.2602 (3)	0.079 (2)	
C14	0.5000 (13)	0.0060 (3)	0.2843 (3)	0.0698 (19)	
C15	0.5497 (11)	0.0725 (3)	0.2485 (2)	0.0558 (17)	
C16	0.1630 (12)	0.0110 (3)	0.0976 (3)	0.0607 (17)	
F2B	0.361 (2)	−0.0028 (6)	0.0569 (4)	0.0788 (14)	0.483 (8)
F3B	−0.057 (2)	−0.0436 (5)	0.0932 (4)	0.0788 (14)	0.483 (8)
H1	0.73080	0.47161	−0.03071	0.0598*	
H4	1.34692	0.19316	−0.10120	0.0768*	
H3	1.08507	0.18001	−0.01281	0.0654*	
H3A	0.40563	0.32185	0.11904	0.0515*	
H6	1.08915	0.42337	−0.12097	0.0737*	
H12	0.12021	−0.09878	0.18408	0.0849*	
H13	0.30519	−0.10167	0.28480	0.0948*	
H14	0.57634	0.00410	0.32511	0.0834*	
H15	0.65944	0.11541	0.26521	0.0668*	
H4A	0.60046	0.13790	0.12033	0.0558*	
H5	1.34620	0.31296	−0.15461	0.0794*	

### Atomic displacement parameters (Å^2^)

**Table:**

	*U*^11^	*U*^22^	*U*^33^	*U*^12^	*U*^13^	*U*^23^
S1	0.0671 (8)	0.0496 (7)	0.0511 (7)	0.0070 (6)	0.0199 (6)	0.0021 (5)
F1	0.086 (2)	0.083 (2)	0.078 (2)	0.0254 (19)	−0.0001 (17)	0.0080 (17)
F2A	0.091 (2)	0.076 (3)	0.070 (2)	0.007 (3)	0.0103 (16)	−0.042 (2)
F3A	0.091 (2)	0.076 (3)	0.070 (2)	0.007 (3)	0.0103 (16)	−0.042 (2)
O1	0.078 (2)	0.0382 (16)	0.0506 (18)	0.0052 (16)	0.0105 (17)	0.0086 (14)
N1	0.063 (2)	0.0381 (19)	0.049 (2)	−0.0037 (17)	0.0070 (18)	0.0155 (16)
N2	0.052 (2)	0.0348 (18)	0.0383 (18)	−0.0034 (16)	0.0060 (16)	0.0034 (15)
N3	0.057 (2)	0.0297 (17)	0.0429 (19)	0.0026 (16)	0.0086 (17)	0.0053 (14)
N4	0.064 (2)	0.0330 (19)	0.045 (2)	−0.0015 (16)	0.0229 (17)	0.0028 (15)
C1	0.049 (2)	0.033 (2)	0.036 (2)	−0.0040 (18)	0.0003 (18)	0.0058 (17)
C2	0.047 (2)	0.042 (2)	0.039 (2)	−0.009 (2)	0.0005 (19)	0.0043 (18)
C3	0.066 (3)	0.048 (3)	0.049 (3)	−0.009 (2)	0.005 (2)	0.003 (2)
C4	0.069 (3)	0.068 (3)	0.058 (3)	−0.008 (3)	0.017 (3)	−0.010 (3)
C5	0.068 (3)	0.085 (4)	0.047 (3)	−0.017 (3)	0.015 (2)	0.003 (3)
C6	0.067 (3)	0.069 (3)	0.050 (3)	−0.015 (3)	0.007 (2)	0.019 (2)
C7	0.045 (2)	0.051 (3)	0.041 (2)	−0.011 (2)	−0.0014 (19)	0.009 (2)
C8	0.055 (3)	0.036 (2)	0.040 (2)	−0.006 (2)	−0.002 (2)	0.0090 (18)
C9	0.047 (2)	0.032 (2)	0.034 (2)	−0.0045 (18)	0.0019 (18)	0.0011 (16)
C10	0.052 (3)	0.031 (2)	0.047 (2)	0.0045 (19)	0.016 (2)	0.0073 (18)
C11	0.057 (3)	0.032 (2)	0.059 (3)	0.005 (2)	0.010 (2)	0.0033 (19)
C12	0.079 (4)	0.033 (3)	0.099 (4)	−0.008 (2)	0.003 (3)	0.013 (3)
C13	0.091 (4)	0.055 (3)	0.093 (4)	0.002 (3)	0.016 (4)	0.038 (3)
C14	0.087 (4)	0.066 (3)	0.057 (3)	0.007 (3)	0.010 (3)	0.023 (3)
C15	0.071 (3)	0.047 (3)	0.050 (3)	−0.003 (2)	0.010 (2)	0.005 (2)
C16	0.066 (3)	0.041 (3)	0.075 (3)	−0.006 (2)	0.004 (3)	−0.007 (2)
F2B	0.091 (2)	0.076 (3)	0.070 (2)	0.007 (3)	0.0103 (16)	−0.042 (2)
F3B	0.091 (2)	0.076 (3)	0.070 (2)	0.007 (3)	0.0103 (16)	−0.042 (2)

### Geometric parameters (Å, °)

**Table:**

S1—C9	1.645 (4)	C2—C7	1.390 (7)
F1—C16	1.340 (6)	C3—C4	1.377 (7)
F2A—C16	1.341 (11)	C4—C5	1.389 (8)
F2B—C16	1.310 (11)	C5—C6	1.373 (8)
F3A—C16	1.354 (10)	C6—C7	1.375 (6)
F3B—C16	1.346 (10)	C10—C15	1.377 (6)
O1—C8	1.229 (6)	C10—C11	1.391 (6)
N1—C8	1.346 (6)	C11—C12	1.384 (7)
N1—C7	1.403 (6)	C11—C16	1.478 (8)
N2—C1	1.286 (5)	C12—C13	1.364 (9)
N2—N3	1.339 (5)	C13—C14	1.362 (8)
N3—C9	1.378 (5)	C14—C15	1.375 (7)
N4—C10	1.420 (5)	C3—H3	0.9300
N4—C9	1.332 (5)	C4—H4	0.9300
N1—H1	0.8600	C5—H5	0.9300
N3—H3A	0.8600	C6—H6	0.9300
N4—H4A	0.8600	C12—H12	0.9300
C1—C2	1.450 (6)	C13—H13	0.9300
C1—C8	1.501 (6)	C14—H14	0.9300
C2—C3	1.384 (7)	C15—H15	0.9300
			
C7—N1—C8	112.0 (4)	C10—C11—C12	119.0 (4)
N3—N2—C1	118.4 (3)	C10—C11—C16	120.9 (4)
N2—N3—C9	120.4 (3)	C11—C12—C13	120.5 (5)
C9—N4—C10	125.3 (3)	C12—C13—C14	120.4 (5)
C8—N1—H1	124.00	C13—C14—C15	120.3 (6)
C7—N1—H1	124.00	C10—C15—C14	120.1 (5)
N2—N3—H3A	120.00	F1—C16—C11	113.3 (5)
C9—N3—H3A	120.00	F1—C16—F2B	113.2 (6)
C10—N4—H4A	117.00	F2B—C16—C11	115.5 (6)
C9—N4—H4A	117.00	F3B—C16—C11	106.3 (6)
N2—C1—C8	127.3 (4)	F2B—C16—F3B	111.4 (7)
N2—C1—C2	126.2 (4)	F1—C16—F3B	95.1 (5)
C2—C1—C8	106.4 (3)	F2A—C16—F3A	106.5 (7)
C1—C2—C7	106.8 (4)	F2A—C16—C11	111.4 (6)
C1—C2—C3	133.1 (4)	F3A—C16—C11	118.7 (6)
C3—C2—C7	120.0 (4)	F1—C16—F2A	94.9 (5)
C2—C3—C4	118.3 (4)	F1—C16—F3A	109.3 (6)
C3—C4—C5	120.8 (5)	C2—C3—H3	121.00
C4—C5—C6	121.5 (4)	C4—C3—H3	121.00
C5—C6—C7	117.4 (5)	C3—C4—H4	120.00
N1—C7—C2	109.2 (4)	C5—C4—H4	120.00
N1—C7—C6	128.8 (4)	C4—C5—H5	119.00
C2—C7—C6	122.0 (4)	C6—C5—H5	119.00
O1—C8—C1	126.7 (4)	C5—C6—H6	121.00
N1—C8—C1	105.7 (4)	C7—C6—H6	121.00
O1—C8—N1	127.6 (4)	C11—C12—H12	120.00
S1—C9—N4	127.4 (3)	C13—C12—H12	120.00
S1—C9—N3	119.4 (3)	C12—C13—H13	120.00
N3—C9—N4	113.2 (3)	C14—C13—H13	120.00
N4—C10—C15	120.0 (4)	C13—C14—H14	120.00
N4—C10—C11	120.3 (4)	C15—C14—H14	120.00
C11—C10—C15	119.7 (4)	C10—C15—H15	120.00
C12—C11—C16	120.0 (5)	C14—C15—H15	120.00
			
C8—N1—C7—C2	−1.7 (5)	C3—C2—C7—N1	179.4 (4)
C8—N1—C7—C6	177.6 (5)	C3—C2—C7—C6	0.1 (7)
C7—N1—C8—O1	−177.0 (4)	C2—C3—C4—C5	0.1 (7)
C7—N1—C8—C1	1.2 (5)	C3—C4—C5—C6	0.3 (8)
C1—N2—N3—C9	179.0 (4)	C4—C5—C6—C7	−0.6 (7)
N3—N2—C1—C2	−178.0 (4)	C5—C6—C7—N1	−178.8 (5)
N3—N2—C1—C8	−2.0 (6)	C5—C6—C7—C2	0.4 (7)
N2—N3—C9—S1	177.9 (3)	N4—C10—C11—C12	179.9 (4)
N2—N3—C9—N4	−2.7 (5)	N4—C10—C11—C16	1.5 (6)
C10—N4—C9—S1	−1.6 (6)	C15—C10—C11—C12	1.0 (7)
C10—N4—C9—N3	178.9 (4)	C15—C10—C11—C16	−177.5 (4)
C9—N4—C10—C11	113.7 (5)	N4—C10—C15—C14	−179.8 (4)
C9—N4—C10—C15	−67.3 (6)	C11—C10—C15—C14	−0.8 (7)
N2—C1—C2—C3	−1.7 (8)	C10—C11—C12—C13	−0.4 (8)
N2—C1—C2—C7	176.0 (4)	C16—C11—C12—C13	178.1 (5)
C8—C1—C2—C3	−178.3 (5)	C10—C11—C16—F1	−56.9 (6)
C8—C1—C2—C7	−0.7 (5)	C10—C11—C16—F2A	48.7 (7)
N2—C1—C8—O1	1.3 (7)	C10—C11—C16—F3A	172.9 (6)
N2—C1—C8—N1	−176.9 (4)	C12—C11—C16—F1	124.6 (5)
C2—C1—C8—O1	177.9 (4)	C12—C11—C16—F2A	−129.8 (6)
C2—C1—C8—N1	−0.3 (5)	C12—C11—C16—F3A	−5.6 (8)
C1—C2—C3—C4	177.1 (5)	C11—C12—C13—C14	−0.4 (9)
C7—C2—C3—C4	−0.3 (7)	C12—C13—C14—C15	0.6 (9)
C1—C2—C7—N1	1.4 (5)	C13—C14—C15—C10	0.0 (8)
C1—C2—C7—C6	−177.9 (4)		

### Hydrogen-bond geometry (Å, °)

**Table:**

Cg1 is the centroid of the C1/C2/C7/N1/C8 ring.

**Table:**

*D*—H···*A*	*D*—H	H···*A*	*D*···*A*	*D*—H···*A*
N1—H1···O1^i^	0.86	2.03	2.864 (5)	163
N3—H3A···O1	0.86	2.09	2.766 (4)	135
N4—H4A···F1^ii^	0.86	2.24	2.998 (5)	147
N4—H4A···F2A	0.86	2.39	2.745 (9)	105
N4—H4A···N2	0.86	2.16	2.582 (5)	110
C3—H3···F3A^iii^	0.93	2.48	3.017 (10)	117
C8—O1···Cg1^iv^	1.229 (6)	3.424 (4)	3.835 (5)	100.1 (3)

Symmetry codes: (i) −*x*+1, −*y*+1, −*z*; (ii) *x*+1, *y*, *z*; (iii) −*x*+1, −*y*, −*z*; (iv) *x*−1, *y*, *z*.
